# Estimation of Median Lethal Concentration of Three Isolates of *Beauveria bassiana* for Control of *Megacopta cribraria* (Heteroptera: Plataspidae) Bioassayed on Solid *Lygus* spp. Diet

**DOI:** 10.3390/insects7030031

**Published:** 2016-06-30

**Authors:** Maribel Portilla, Walker Jones, Omaththage Perera, Nick Seiter, Jeremy Greene, Randall Luttrell

**Affiliations:** 1 Southern Insect Management Research Unit, Agricultural Research Service (ARS), USDA, 141 Experiment Station Road, Stoneville, MS 38776, USA; op.perera@ars.usda.gov (O.P.); randy.luttrell@ars.usda.gov (R.L.); 2 National Biological Control Laboratory, Agricultural Research Service (ARS), USDA, 59 Lee Road, Stoneville, MS 38776, USA; walker.jones@ars.usda.gov; 3 Southeast Research and Extension Center, Division of Agriculture Sciences and Natural Resources, University of Arkansas, P.O. Box 3508, Monticello, AR 71656, USA; nseiter@uaex.edu; 4 Department of Agricultural and Environmental Sciences, College of Agriculture, Forestry, and Life Sciences, Clemson University, Edisto Research and Education Center, 64 Research Road, Blackville, SC 29817, USA; greene4@clemson.edu

**Keywords:** Kudzu bugs, *Beauveria bassiana*, NI8, GHA, *Megacopta cribraria*, biological control, solid diet

## Abstract

The kudzu bug, *Megacopta cribraria* (F.), is an urban nuisance and significant agricultural pest. The median lethal concentrations of three strains of *Beauveria bassiana* (Balsamo), including the Mississippi Delta native strain (NI8) isolated from *Lygus lineolaris* (Palisot de Beauvois), the commercial strain BotaniGard^®^ (GHA) (Victor, NY, USA), and the *B. bassiana strain* isolated from *M. cribraria* (KUDSC), were estimated on kudzu bug adults. A technique developed to evaluate *B. bassiana* against *L. lineolaris* was used. Younger adults (eight days after collection) were treated with NI8 and GHA and older adult (50 days after collection) were treated with NI8, GHA and KUDSC. Higher concentrations (*n* × 10^6^, *n* × 10^7^) of NI8 and GHA caused kudzu bug mortality two days after treatment in younger adults and similar concentrations of NI8, GHA, and KUDSC caused mortality one day after treatment in older adults. Lower concentrations (*n* × 10^4^, *n* × 10^5^) were not significantly different in mortality between strains. LS_50_ values of the KUDSC were significantly lower than NI8 and GHA values in older adults. This is the first available information on median lethal concentration of *B. bassiana* on kudzu bug adults bioassayed on artificial diet. It was determined that *B. bassiana* (KUDSC and NI8) are highly effective for young adults at very low doses (LC_50_ 1.98–4.98 viable spores per mm^2^).

## 1. Introduction

The kudzu bug, *Megacopta cribraria* (F.) (Hemiptera: Plataspidae), is an urban nuisance and a significant agricultural pest. Native to Asia and the India subcontinent [1], *M. cribraria* was first discovered in the U.S. in 2009 feeding on kudzu, *Pueraria montana* (Lour) Merr. (Fabaceae). It was subsequently found congregating in large numbers on the exteriors of nearby homes and vehicles in nine counties in northeastern Georgia [2]. In 2011, its geographic range continued to increase, moving rapidly to cover much of North Carolina, South Carolina, Alabama, Mississippi and Florida [3]. In 2012, the distribution expanded northwest into central Virginia [4]. During the following spring and summer, the insect was detected in eight counties in southern Maryland and the eastern Shore, as well as the District of Columbia [5].

*M. cribraria* feeds primarily on leguminous plants such a kudzu and soybean (*Glycine max* Merrill). *M. cribraria* has caused serious damage to soybean in central and southern China [1,6] when both nymphs and adults feed by piercing and sucking stem and leaf tissue, causing reductions in photosynthesis and stunting the growth of the plants [1,7]. Although, *M. cribraria* has not been reported to feed on seed pods, the combination of stem and foliar damage, and reduced photosynthesis from sooty mold, leads to leaf discoloration, deformation of pods, and reduction in seed size [7]. Recently, *M. cribraria* has become a major pest of soybean in the southern U.S., where yields can be reduced by up to 60%, due to decreases in numbers and weight of seeds [8]. This insect has the potential to become a major pest in Maryland [5]. Populations of kudzu bugs can be difficult to suppress because of their highly mobile nature, despite the availability of insecticide treatment of individuals [9]. Few natural enemies of kudzu bug have been observed in the U.S. [10,11,12]. This likely contributes to the insect’s rapid geographic expansion [13]. Additional information is needed to identify effective alternative management options.

The entomopathogenic fungus *Beauveria bassiana* infects kudzu bugs in Asia [14]. However, no data on *B. bassiana* pathogenicity and subsequent mortality are available from its U.S. range [15]. In 2012, cadavers of adults of *M. cribraria* infected with *B. bassiana* were found on soybean plants at the Clemson University, Edisto Research and Education Center in Blackville, South Carolina, SC, USA [8]. Infected insects from those samples were sent to the USDA-ARS Southern Insect Research Unit in Stoneville, Mississippi, MS, USA for identification and additional study. The main objectives of this research were to isolate and identify the entomopathogenic fungus from *M. cribraria* and estimate the lethal concentration and doses comparing its infectivity in the laboratory using *Lygus* artificial diet against two known pathogenic isolates, the Mississippi Delta strain NI8 known for its high virulence to *Lygus lineolaris* (Palisot de Beauvois) [16,17,18,19,20] and the commercial strain GHA [6,20,21,22]. Results demonstrated that bioassay chamber spray and protocol on artificial diet developed for *L. lineolaris* was an efficient method that could be employed to characterize the pathogenicity of *B. bassiana* against young and old adults of *M. cribraria*. The results showed the capacity of *M. cribraria* to acquire lethal doses of conidia (asexual reproductive spore) from the delivery spray facilitating the estimation of LD_50_ (Lethal Dose) and LS_50_ (Letha Sporulation) of this well-known entopathogenic fungus. The importance of sporulation in this study was a key to ensure the survival of the conidia under the conditions that the insect and fungus were faced. Based in this result it is assumed that *B. bassiana* could impact *M. cribraria* population, suppressing this insect before colonizes soybean.

## 2. Materials and Methods

### 2.1. Colonies of Megacopta cribraria

Adults of *M. cribraria* were collected in October of 2012 from late-season soybeans at the Clemson University Edisto Research and Education Center in Blackville, South Carolina (latitude 33.356305° N, longitude 81.304693° W). Collected insects were sent and held in a greenhouse at the Stoneville Research Quarantine Facility (SRQF) at USDA-ARS National Biological Control Laboratory in Stoneville, Mississippi, MS, USA. Insects were maintained in screened cages 60 cm^3^ at 27 °C and fed kudzu, *P. montana* for 50 days. Fresh plant material was collected when necessary from a small patch of kudzu vine naturally growing near the SRQF in Stoneville, Mississippi, MS, USA.

### 2.2. Fungal Isolates and Identification of KUDSC

Cadavers of kudzu bugs with external signs of fungal growth were collected from soybean plants during early October 2012 at the Clemson University Edisto Research and Education Center in Blackville, South Carolina, SC, USA and sent to SRQF in Stoneville, Mississippi, MS, USA. Conidia from infected insects were obtained by scraping the infected insects and suspending the resulting fungal material in 5 mL of 0.04% Tween^®^ 80 (Fisher Scientific, CAS 9005-65-6, St. Louis, MO, USA). Aliquot (100 μL) of the resulting suspension were pipetted onto plate of Sabouraud Dextrose Agar Premix (SDAY) (10 g/L Peptone, 40 g/L Dextrose, 15 g/L Agar, 2 g/L Yeast Extract) and incubated for 24 h at 27 °C. Single spores collected from individual plate of each strain were used for identification by morphological characteristics [23]. For molecular identification, genomic DNA was extracted from one of the 4-day old single-spore cultures using MasterPure genomic DNA extraction kit (Epicentre, Madison, WI, USA). Sections of mitochondrial cytochrome oxidases 1 (*cox1*), *cox3*, and NADH dehydrogenase 4 (*nad4*) were PCR amplified using primer pairs 1397F/1398R (5’-GATTAGAATTAAGTGGACCAGGAGTTCA-3’/5’-GCTAAAACAACACCACTTAATCCTCCTA-3’), 1389F/1390R (5’-AACAAGAAATCATTTTCAAGATCATCCT-3’/5’-GCTTCATAACCTAAATGGTGATGATC-3’), and 1393F/1394R (5’-TAAAAGCTCATGTTGAAAGTCCTTTAGG-3’/5’-GAACCACCAAATGCTGTTCTATTAAACA-3’), respectively. The amplicon sizes expected from *cox1*, cox3, and *nad4* were 964, 724, and 614 bp, respectively. Each amplification was performed in a 25 μL reaction volume in 1× LongAmp buffer (New England Biolabs, Ipswich, MA, USA) containing 60 mM Tris-SO_4_, 20 mM (NH4)_2_SO_4_, 2 mM MgSO_4_, 3% Glycerol, 0.06% IGEPAL^®^ (SIGMA-ALDRICH, St. Louis, MO, USA) CA-630, 0.05% Tween^®^ 20 (pH 9), 0.4 mM dNTP, 200 nM of gene specific forward and reverse primers, and 1 unit of LongAmp *Taq* polymerase. Thermal cycling was performed on a PTC100 thermal cycler (BioRad, Laguna Hills, CA, USA) with an initial denaturation step of 1 min at 95 °C followed by 35 cycles of 15 s at 95 °C, 10 s at 52 °C, and 60 s at 72 °C. Amplification of targets were verified by agarose gel electrophoresis and the amplicons were cloned into pCR2.1 T-A cloning vector (Life Technologies, Grand Island, NY, USA). Nucleotide sequences of eight recombinant clones per amplicon were obtained by Sanger dideoxy sequencing at the USDA-ARS Genomic and Bioinformatics Research Unit, Stoneville, Mississippi. Sequences were visualized and curated using Vector NTI v11.5 software (Life Technologies, Grand Island, NY, USA) and identity of *cox1*, *cox3*, and *nad4* genes were validated by searching public nucleotide databases available at the National Center For Biotechnology Information (NCBI) and aligning with the mitochondrial genome of *B. bassiana* (accession: EU37153). Validated nucleotide sequences were submitted to GenBank.

Nucleotide sequences of eukaryotic translation elongation factor 1 alpha (EF-1α) and internal transcribed spacer 1 and 2 of ribosomal RNA (ITS) were obtained using [24]. Nucleotide sequence reads were used to search databases to identify similarity to EF-1α and ITS sequences from other *B. bassiana* isolates.

### 2.3. Production of KUDSC Spore Powder

The Mississippi Delta native strain NI8 isolated from *L. lineolaris* and the commercial strain GHA formulated as BotaniGard^®^ were obtained from stored sources of spore powder maintained at the USDA-ARS Southern Insect Research Unit (SIMRU). NI8 and GHA are produced at SIMRU in a regular basis for the *L. lineolaris* research program, stored sources are kept at −80 °C. To produce KUDSC spore powder, a small-scale of biphasic culture system for solid-substrate fermentation was used according to the method used at SIMRU as follow: a singe germinated spore was selected from a plate (plate used for identification above) manually with a toothpick and suspended in 500 mL of CSYE broth (40 g /L Glucose, 10 g/L KNO_3_, 5 g/L KH_2_PO_4_, 2 g/L MGSO_4_, 0.05 g/L CACL_2_, 2.5 g/L Yeast Extract). The suspension was agitated in a shaker (Incubator Shaker Series Excella E25, New Brunswick Scientific Co., Inc., Edison, NJ, USA) for three days at 25 °C. Ten plastic bags (PPB75SEH6/V35-53 580 mm × 385 mm × 360 mm) that contained 1000 mL of barley (Minnesota Grain Inc., SASO, East Grand Fork, MN, USA) and 600 mL of water were sealed, autoclaved and prepared as production medium. Aliquots of 50 mL of the inoculum (suspended *B. bassiana* spores-CSYE broth) were injected into each bag and placed in an environmental control room (27 °C, 85% RH, 12 D:12 L Photoperiod) for 10 days and allowed to ferment. The injected bags were routinely shaken to avoid coagulation or clusters allowing for optimal dispersion of the inoculum throughout the fermented substrate. The sporulated substrate was transferred to paper sacks (30.48 cm × 17.78 cm × 43.18 cm Barrel, Kraft) for ten more days or until the moisture content of the resulting conidia dried to a_w_ ≤ 0.3. Moisture endpoint was measured using a water activity meter (AquaLab-Decagon Devices, Serie 3-0105641113; Decagon Devices Inc., Pullman, WA, USA). Conidia were separated from the dried substrate using graded sieves (Grainger, Sieve SS Frame 8, SS Mesh # 30 and 100) on a vibratory shaker (Advantech, 8 Test Sieve Shaker, Model Mainll-16Y908; Endecotts Limited, London, UK). Harvested spore powder from the KUDSC strain and samples of NI8 and GHA were examined for spore germination and spore quantification (spores mm^2^). Amounts of 0.5 g of harvested spore powder that contained 1.20 × 10^11^, 1.18 × 10^11^, and 1.19 × 10^11^ spores per gram for NI8, KUDSC, and GHA, respectively were suspended in 50 mL of 0.04% Tween-80 (Sigma-Aldrich P8074) and diluted to obtain final concentrations of 7.02 × 10^7^, 6.95 × 10^7^, and 6.90 × 10^7^ spores per mL, respectively. Suspensions of conidia were sprayed on five disposable microscope cover slips using a spray tower modified from a Burgerjon tower [19] that covered an area of 38.5 cm in diameter. Concentrations (spores per mm^2^) were quantified by counting spores deposited [19]. The process was replicated five times using the final concentrations (*n* × 10^7^) (*n* = diluted harvested spore powder/strain: 7.02, 6.95, 6.90 for NI8, KUDSC and GHA, respectively) prepared above. Resulting data were analyzed by analysis of variance [25]. Lower test concentrations (*n* × 10^6^, *n* × 10^5^, *n* × 10^4^) were extrapolated based on dilution of *n* × 10^7^ concentrations, and the number of spores applied was corrected for viability (germination) [26] for all concentrations (Table 1). Aliquots of 6 mL of suspension (*n* × 10^7^) of each strain provided concentrations of about 350 viable spores per mm^2^ (Table 1).

### 2.4. Bioassay Procedure

Serial dilutions of four test concentrations of NI8, GHA, and KUDSC strains (*n* × 10^7^, *n* × 10^6^, *n* × 10^5^, *n* × 10^4^ spores/mL) were prepared to treat kudzu bug adults and evaluate mortality and infection. Kudzu bug adults (unknown age) from the South Carolina collection site were separated into two groups. The first group (called young adults) was sprayed with NI8 and GHA 8 days after field collection on 8 October 2012. The second group (called older adults) were held for 50 days and sprayed with NI8, GHA, and KUDSC strains on 20 November 2012. The KUDSC strain was not applied to the young adults, as that strain was received and isolated after the first group was treated. Both bioassays were conducted at room temperature (23 ± 2 °C and 50% ± 5% RH). Each assay treatment (concentration of individual strain) was replicated four times with 10 adults per treated replicate (380 and 520 individual per young and old adult groups, respectively). Control insects were sprayed with 6 mL water (water control). Treatments of NI8, GHA, and KUDSC (*n* × 10^7^, *n* × 10^6^, *n* × 10^5^, *n* × 10^4^ spores/mL) concentrations (Table 1) were similarly delivered in a 6 mL spray volume. After application, insects were placed individually into a 29.7 mL SOLO cups with a solid diet developed for use in *L. lineolaris* bioassays [19]. RH of 80% within a diet solo cup (bioassay arena) has been reported [19]. Adults were examined daily for 10 days for mortality and sporulation. Dead insects were retained in the same cup until completion of the 10 days trial to observe sporulation. Sporulation percentage was measured for cadavers on which sporulation occurred (presence of mycelial growth) out of total dead.

### 2.5. Statistical Analysis

Computations for all experiments were performed using SAS system software [25]. A randomized complete block design with factorial arrangements was used for each group of insects as follow: 2 × 5 × 3 (young adults) and 3 × 5 × 3 (old adults) for mortality (strains: NI8 and GHA; concentrations: *n* × 10^7^, *n* × 10^6^, *n* × 10^5^, *n* × 10^4^ spores/mL; and evaluation times: 3, 5, and 10 days after sprayed) and 2 × 5 (young adults) and 3 × 5 (old adults) for sporulation (strains: NI8, GHA, and KUDSC; and concentrations: same as above). Each treatment combination was repeated four times. Nonparametric estimates of the survival function of kudzu bugs were compared between treatments using PROC LIFETEST [25]. Statistical differences in the survival of *M. cribraria* were declared based on the log-rank statistic. Mortality and infection were analyzed by using PROC GLM to detect differences between treatments for each group of insects. Mortality and sporulation data for each group of insects and each strain were analyzed by PROBIT [25] using common logarithm (log to the base 10) of the concentration value.

## 3. Results

### 3.1. Verification of Identity of Fungal Isolate KUDSC

Cloned amplicons yielded nucleotide sequences cox1 (KR733105.1), cox3 (KR733106.1), and nad4 (KR733107.1), EF-1α (KX228572), and ITS (KX228573). Searches of BLAST databases using these nucleotides revealed highest nucleotide identity (>99%) to respective mitochondrial genes of *B. bassiana* (accessions: EU37153.1 and EU100742.1). These nucleotide sequences were less than 95% identical to the entomopathogenic fungi *Lecanicillium muscarium* Zare and Gams (Hypocreales: Clavicipitaceae) (AF487277.1) and Cordyceps militaris, L. (Hypocreales: Cordycipitaceae) (KF432176.1). Therefore, it was determined that the KUDSC isolates used in this study were a strain of *B. bassiana*. Database searches matched *M. cribraria* EF-1α sequence with that of *B. bassiana* isolate 320 (AY531926.1) at 99.94% identity (one mismatch out of 1710 nucleotides). ITS sequence of the same *B. bassiana* isolate (AY532018.1) matched 571 of the 573 nucleotides of *M. cribraria* ITS sequence (99.65%). The next closest match was to *B. bassiana* isolate 252. The EF-1α (AY531913.1) and ITS (AY532004.1) sequences of the *B. bassiana* isolate 252 matched *M. cribraria* EF-1α and ITS sequences at 99.71 and 99.65%, respectively. Phylogenetic analysis of combined EF-1α and ITS sequences matched the *B. bassiana* isolates of Clade A [24]. All nucleotide sequence analyses indicated that the *B. bassiana* recovered from *M. cribraria* was a novel isolate.

### 3.2. Dose-Mortality Response of M. cribraria to B. bassiana

**Figure 1:**
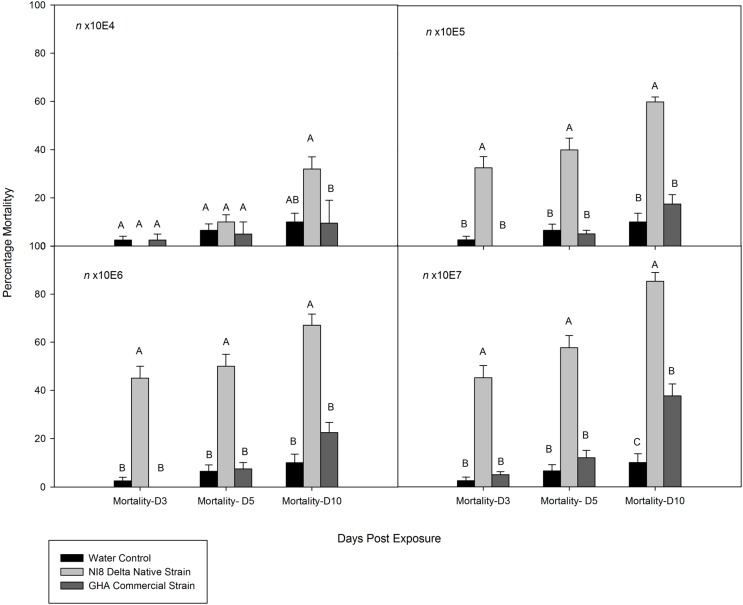
Cumulative mortality percentage of young adults (treated eight days after collection) of *Megacopta cribraria* exposed to *Beauveria bassiana* at different concentrations under laboratory conditions. Insects were fed with artificial diet after spray. Columns within the group labeled with a different letter were significantly different at *p* = 0.05 (Tukey test).

**Figure 2:**
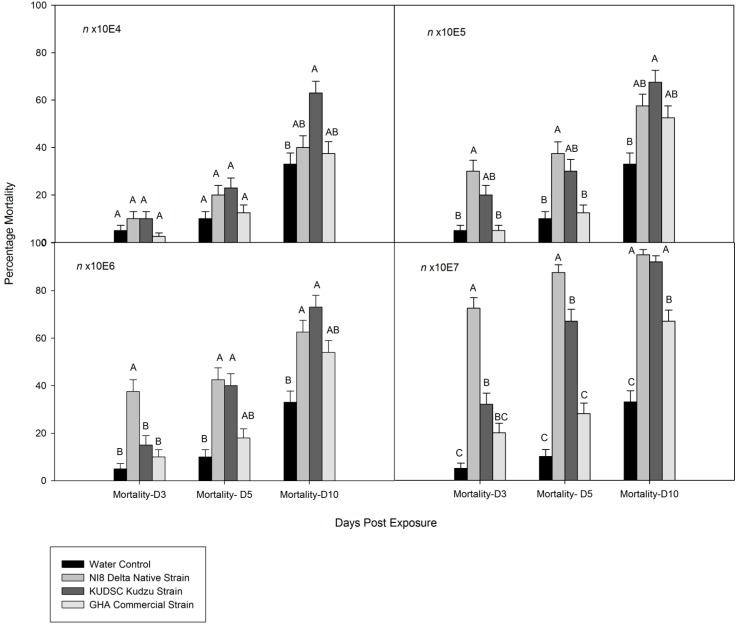
Cumulative mortality percentage of old adults (treated 50 days after collection) of *Megacopta cribraria* exposed to *Beauveria bassiana* at different concentrations under laboratory conditions. Insects were fed with artificial diet after spray. Columns within the group labeled with a different letter were significantly different at *p* = 0.05 (Tukey test).

All isolates tested were pathogenic to kudzu bug adults. However, the mortality among isolates was highly variable (Figure 1 and Figure 2). The GHA commercial strain had the lowest measured performance at all concentrations at all evaluation times. No significant differences in mortality were found between the KUDSC and NI8 strains at the highest concentrations tested (n × 10^6^ and n × 10^7^) for studies with old adults at 10 days after treatment (Figure 2). Mortality in the first group with younger adults exposed to NI8 was three- to eight-fold greater than that of GHA, but no significant differences were found in sporulation between strains at all concentrations except the n × 10^6^ (*F* = 6.37; df = 3, 119; *p* = 0.0024). Mortality at three and five days for young adults (*F* = 0.50; df = 3, 119; *p* = 0.6105 and *F* = 0.91; df = 3, 119; *p* = 0.4042) and old adults (*F* = 0.91; df = 3,159; *p* = 0.4382 and *F* = 1.11; df = 3, 159; *p* = 0.3453) exposed to the lowest concentrations (n × 10^4^) of all strains was not significantly different from those insects exposed to water. Results from analyses of dose–mortality response by insect age and dose ratio for the three isolates indicated high variability in dose–mortality responses between adult age groups (Table 2). Dose-ratios were higher for young adults than those for older adults. Isolate GHA was less virulent than NI8 in young adults. At 7.02 × 10^7^, NI8 strain eventually killed about 60% while GHA eventually killed only about 40% of the insects. Comparison of LD_50_ values among the three isolates in older adults did not show any significant differences among strains.

### 3.3. Time-Mortality Response of M. cribraria to B. bassiana

**Figure 3:**
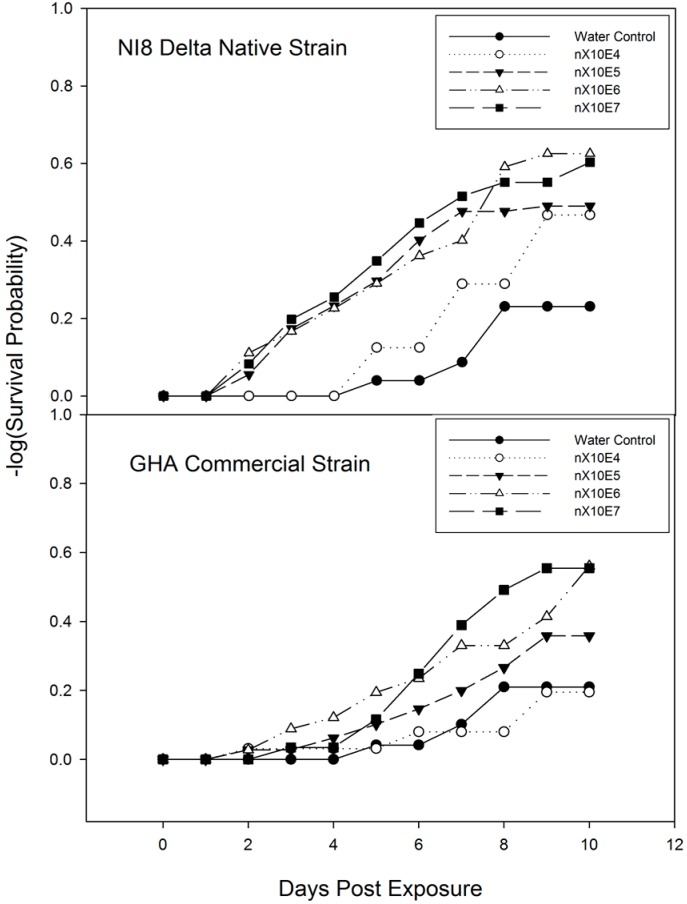
Survival probability of young adults (treated eight days after collection) of *Megacopta cribraria* exposed to *Beauveria bassiana* at different concentrations under laboratory conditions. Insects were fed with artificial diet after spray. *p* = 0.05, LIFETEST of Equality Over Strata.

**Figure 4:**
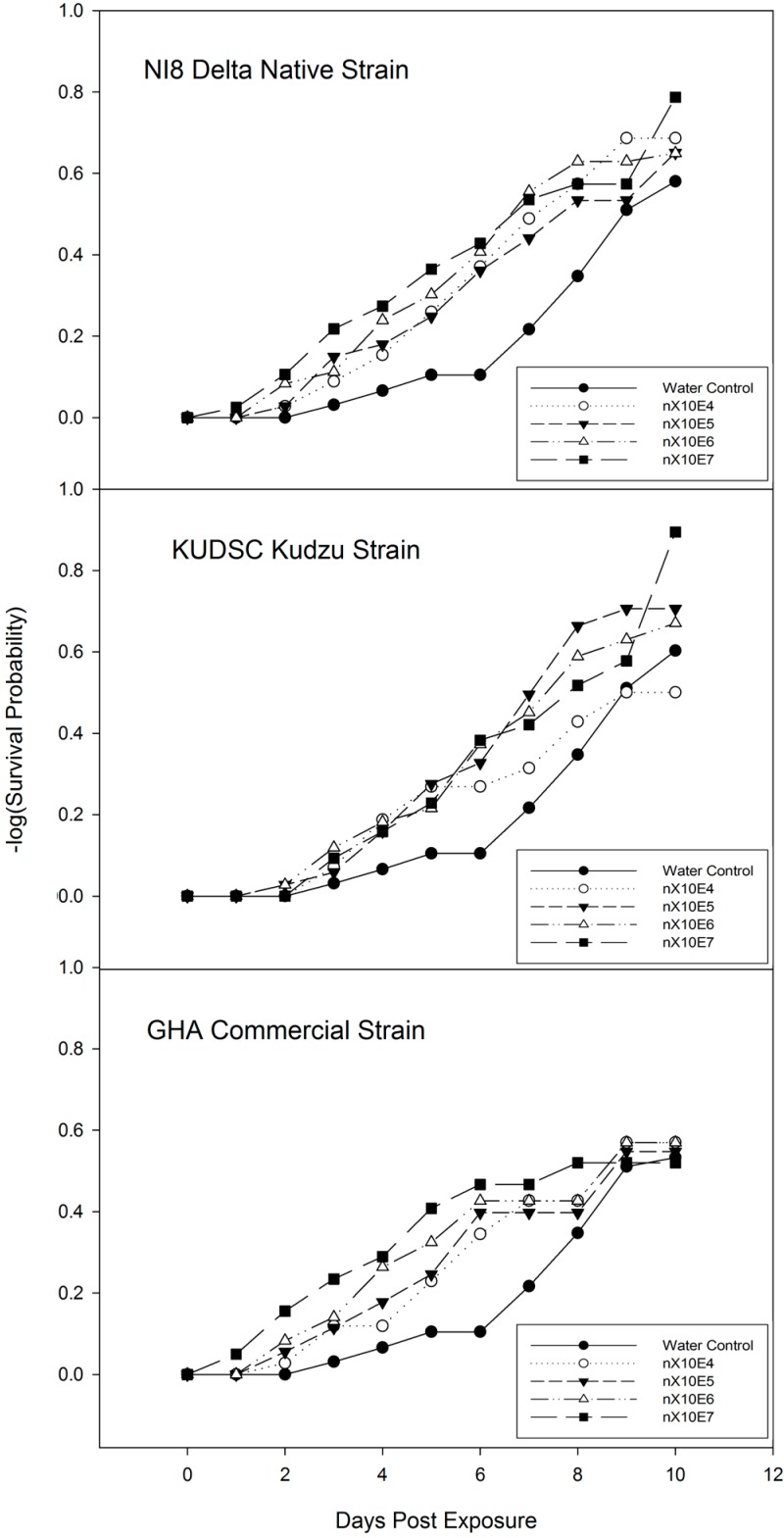
Survival probability of old adults (treated 50 days after collection) of *Megacopta cribraria* exposed to *Beauveria bassiana* at different concentrations under laboratory conditions. Insects were fed with artificial diet after spray. *p* = 0.05, LIFETEST of Equality Over Strata.

Time to mortality was measured through routine post-treatment observations. Higher rates plateaued faster than lower rates (Figure 3 and Figure 4). The earliest mortality recorded was with the highest concentration of both *B. bassiana* strains on two and three days after treatment in young adults (Figure 3) while older insects treated with NI8 and KUDSC strains began to exhibit mortality at one and two days after treatment (Figure 4). Mortality analyzed by the test of equality with the strata statement in −log (survival probability) PROC LIFETEST indicated no significant differences between concentrations for all isolates, with the exception of younger adults treated with NI8 (Log-Rank X^2^ = 11.67, df = 4, *p* = 0.019) (Figure 3 and Figure 4).

### 3.4. B. bassiana Dose-Sporulation response (LS_50_) on M. cribraria: Effects of Strain and Concentration

**Figure 5:**
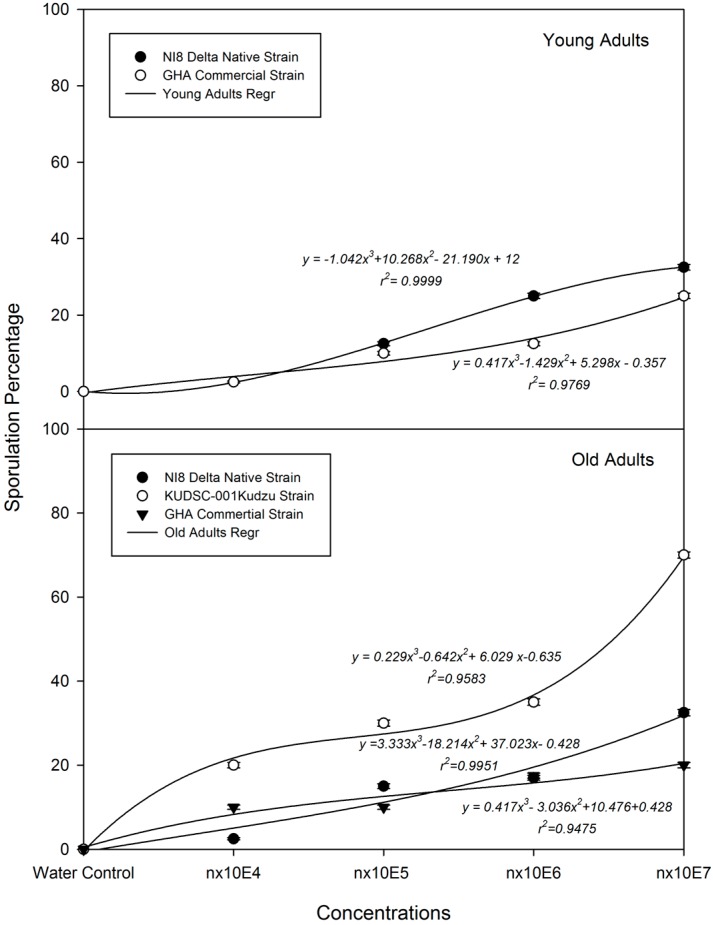
Linear regression (GLM) analysis predicting probability percentage of *Beauveria bassiana* sporulation on young and old adults of *Megacopta cribraria* as a function of the dose level.

The regression analysis performed (cubic trend model) with sporulation percentage per concentration, showed significant correlation between sporulation and spore concentration (Figure 5). Sporulation increased when spore concentrations increased, ranging the r^2^ values from 0.94 to 0.99. The sporulation of the KUDSC strain was significantly higher than GHA and NI8 at all concentrations: *n* × 10^4^ (*F* = 4.56, 0.81; df = 3, 3, 159; *p* = 0.0043, 0.4897), *n* × 10^5^ (*F* = 5.70, 0.99; df = 3, 3, 159; *p* = 0.0010, 0.4001), *n* × 10^6^ (*F* = 6.36, 0.43; df = 3, 3, 159; *p* = 0.0004, 0.7286), and *n* × 10^7^ (*F* = 22.47, 0.27; df = 3, 3, 159; *p* < 0.0001, 0.8483). Sporulation percentage of KUDSC was 2.0-, 2.0-, 2.1-, and 2.2-fold greater than NI8 and 2.0-, 3.0-, 2.1-, and 3.5-fold greater than GHA at *n* × 10^4^, *n* × 10^5^, *n* × 10^6^, and *n* × 10^7^, respectively. Analyses of dose-sporulation response by insect age and dose-ratio for all three isolates showed high variability in dose-sporulation response among adult age groups (Table 3). Dose-ratios for sporulation response (LS_50_) were significantly higher for young kudzu adults than those ratios found for older adults. Isolate GHA presented less sporulation that NI8 in young adults and KUDSC showed a significantly higher infectivity in old adults among NI8 and GHA strains.

## 4. Discussion

The original goal for this study was to determine the lethal effects of NI8 and GHA strains on populations of adult of kudzu bug that would soon be moving to overwinter sites. The addition of the KUDSC strain to this investigation provided an opportunity to evaluate the lethal effects of this naturally occurring strain on populations of kudzu bug adults and compared its activity to NI8 and GHA.

The significant differences in percent mortality among the strains, indicates that young and old adults of *M. cribraria* can readily acquire lethal doses of conidia from the direct inoculation method (spray delivery) developed for *Lygus spp*. [19]. The lack of significant differences in –log survival probability (Figure 3 and Figure 4) shows that the *Lygus spp*. diet was not the optimal diet for *M. cribraria*. This likely accounts for the mark increase in mortality noted on Days 6 and 8 in the water-control. However, considering the results, the young and old adult controls survive long enough (>10% mortality) on the solid diet to facilitate the comparison of pathogenesis and sporogenesis phases against strains and concentrations. Infection of *B. bassiana* spores normally is wrapped with haemocytes, but after four days only the germ tube breaks through its envelope and invades the insect’s hemolymph [27]. Other observation also indicated that a five-day incubation period at 25 °C is adequate for detection of most lethal infections [28]. In this study, mortality and sporulation are the main factor of evaluation to determine virulence (level of pathogenicity) of *B. bassiana* against *M. cribraria*.

The entomopathogenic fungus *B. bassiana* is known to infect kudzu bug in Asia [14]. Few reports of pathogenicity and subsequent mortality are available from its recent establishment in the U.S. where *B. bassiana* infection in caged plots of soybean with high densities of *M. cribraria* has been observed [8]. Two *B. bassiana* infected kudzu bug specimens were also reported in Georgia [13]. Recently, high incidences of *B. bassiana* were observed in collections of kudzu bug nymphs and adults from kudzu growing in Mississippi and Tennessee [29]. Infection rates estimated in these studies ranged from 10% to 95%. However, only limited published data on the lethal effects of *B. bassiana* on kudzu bug are available and no studies of bioassays on artificial diet has been reported.

The entomopathogenic fungus *B. bassiana* is a globally distributed, monophyletic taxon that has been isolated from a wide range of insect taxa [24]. Selection of virulent genotypes is an important aspect of enhancing microbial control of insects with entomopathogenic fungi [30]. The selection may increase the ability to sporulate on the host cadaver. The isolations of the KUDSC strain from *M. cribraria* in South Carolina and NI8 from *L. lineolaris* in the Mississippi Delta are just examples of virulent genotypes with high pathogenicity against targeted pests.Tthe highest concentration of NI8 used in this study (*n* × 10^7^) has been enough to obtain 100% mortality and 97% sporulation in *L. lineolaris* under laboratory conditions 10 days after spray [19]; but in this study, although more than 85% and 95% mortality was obtained in young and old adults respectively, only 32.5% ± 4.7% (SD) and 35.0% ± 4.8% (SD) sporulation was observed on treated young and old adults of *M. cribraria*, respectively. The NI8 strain of *B. bassiana* showed high pathogenicity to old adults of *M. cribraria,* but not significantly more mortality than that of the KUDSC strain at the highest test concentrations (*n* × 10^6^ and *n* × 10^7^) (Figure 2). Young and old kudzu bug adults treated with the commercial strain GHA showed low mortality and sporulation at all concentrations (Figure 1, Figure 2 and Figure 5). Similar results have been observed in several studies using commercial isolates of *B. bassiana* where moderately effective control of targeted insects was reported [18,21,31,32,33,34]. The present results support previous observations that pathogenicity of *B. bassiana* is not always related to the original host or geographical origin [33,35,36,37,38,39,40,41,42].

One disadvantage of entomopathogenic fungi is the long time period that elapses between exposure and death of the host [39]. The time period between exposure and mortality in this investigation ranged from 1 to 8 days for the highest and lowest concentration tested, respectively. These time periods of mortality do not differ from studies previously reported. For example a median lethal time of 4.9–8.4 days on adults and nymphs was observed on *L. lineolaris* [19], 4.0–5.7 days on *Leptocorisa oratorious* F. (Hemiptera: Alydidae) [43], 4–8 days on *Coptotermes formosanus* Balsamo and *Reticulitermes flavides* Kollar (Isoptera: Rhinotermitidae) [6] and 10.7 days on *Nezara viridula* L. (Hemiptera: Pentatomidae) [44]. For standard viability determination, *B. bassiana* conidia are typically incubated from 16–24 h on agar-based media. Germination on the insect integuments should be slower, due to limited free moisture and the time required for penetration. In this study sporulation was observed 2–6 days after insect mortality for all strains at all concentrations. However, the successful accomplishments of fungal growth, which are pre-requisites for pathogenicity, were primarily observed in old adults sprayed with the KUDSC strain (Figure 5). Sporulation percentage of cadavers treated with KUDSC was 2.2-fold greater than those for NI8 and 3.5-fold greater than those for GHA at the highest test concentration.

Estimating the media lethal dose of the fungal pathogen *B. bassiana* for *M. cribraria* acquired via spray acquisition of conidia is a process that has not been attempted; particularly on artificial diet. Estimating the LD_50_ and LS_50_ for *M. cribaria* requires not only a reliable method to evaluate the infection of propagules on its body, but also a determination of when to sample the individuals exposed to the inoculum. In the described bioassay, dose-ratios for mortality and sporulation were significantly higher for old adults of *M. cribraria* than those ratios found for young adults. The high susceptibility obtained in old adults may be occurred due to the low mobility of those insects at the time of the spray, assuming that its low activity could probably let them acquire more conidia than that on young adults, which effectively contributed to its infection, mortality and sporulation. In this experiment Conidial acquisition was a continued process (spray delivery) for a same period of time of the exposure for old and young adults while treated insects were in movement.

## 5. Conclusions

This study resulted in a reliable bioassay system that determined the median lethal concentration and sporulation of *B. bassiana* against young and old adults of *M. cribraria*. The LD_50_ of 0.83 spores/mm^2^ and LS_50_ of 64.38 spores/mm^2^ obtained for the KUDSC strain could have great potential for controlling late-season populations of kudzu bug. Compared with NI8 LC_50_s and LS_50_s for KUDSC were 5.2-fold and 238-fold lower, respectively. Although no results are presented in this study for mortality and sporulation caused by the KUDSC strain on early season young adults, it is assumed that LD_50_ and LS_50_ values (spores/mm^2^) would be a little greater than those values observed for late-season adults. High infection of kudzu bug has been observed in Mississippi and Tennessee (MS, TN, USA), which is suggesting that the *B. bassiana* strains NI8 (ARSEF13136) and KUDSC (ARSEF13137) may provide useful levels of suppression and a potential bio-control option for kudzu bug in soybean [29]. These experiments were all conducted in the laboratory under conditions that are favorable to *B. bassiana*. Further studies are needed that examine the pathogenicity of these two strains of *B. bassiana* on various insect pests in soybeans field plots. In general the study reported here resulted that the bioassay system developed for *L. lineolaris* [18] was also successful to bioassayed *M. cribraria*. The current limitation of *M. cribraria* survival and development on this diet can be improved by modifying the existing *Lygus diet*. However, this bioassay system has been used to easily set up and manipulate first to fifth instar nymphs of *M. cribraria*, where the survival nymphs molted to the next instar producing data that showed good fit to the classical probit model for each instar [29].

## Figures and Tables

**Table 1:** Germination and Spores Quantification of three *Beauveria bassiana* Strains.

Strain	Spores/g	Spores/mL	Spores/mm^2^ (n ± SD)	
			Total	Viable
NI8 Native Delta Strain	1.20 × 10^11^	7.02 × 10^7^	395 ± 107 ^a^	375 ± 100 ^a^
GHA Commercial Strain	1.18 × 10^11^	6.95 × 10^7^	356 ± 74 ^a^	339 ± 69 ^a^
KUDSC South Carolina Kudzu Strain	1.19 × 10^11^	6.90 × 10^7^	365 ± 97 ^a^	347 ± 91 ^a^

**Table 2:** Dose-mortality response (LD_50_) of young and old adults of *Megacopta cribraria* treated with tree strains of *Beauveria bassiana* applied at four concentrations.

Insect Age—Strains	Dose-Mortality Response (spores/mm^2^)						
	Slope ± SE	LC_50_ (95% CI)	Probit Trend				Dose-Ratio
			Test for Slope ^1^		Test for GoF ^2^		
			X^2^	*p* > X^2^	X^2^	*p* > X^2^	
Young—NI8 ^3^	0.207 ± 0.044	4.989 (0.312—40.945)	12.72	0.0004	22.08	<0.0001	1
Young—GHA ^4^	0.186 ± 0.069	4663 (427.812—5.885^10)	7.27	0.0070	0.977	0.4652	934
Old—NI8	0.241 ± 0.048	4.363 (0.404—26.356)	25.61	<0.0001	0.918	0.521	0.87
Old—GHA	0.119 ± 0.043	1.979 (0.0006—84.391)	7.53	0.0061	0.131	0.9997	0.40
Old—KUDSC-001 ^5^	0.117 ± 0.041	0.830 (0.0001—32.827)	7.81	0.0052	1.529	0.113	0.20

^1^ Test for slope—significance indicates dose affects mortality. ^2^ Test for Goodness of Fit (GoF) significance indicates error from Probit trend is greater than expected for simple binomial response. ^3^ NI8-Native Delta Nississippi Strain (ARSEF 8889). ^4^ GHA—Commercial strain. ^5^ KUDSC-001—South Carolina Kudzu Strain (ARSEF 13136).

**Table 3:** Dose-sporulation response (LS_50_) of young and old adults of *Megacopta cribraria* treated with three strains of *Beauveria bassiana* applied at four concentrations.

Insect Age—Strains	Dose-Sporulation Response (spores/mm^2^)						
	Slope ± SE	LS_50_ (95% CI)	Probit Trend				Dose-Ratio
			Test for Slope ^1^		Test for GoF ^2^		
			X^2^	*p* > X^2^	X^2^	*p* > X^2^	
Young—NI8 ^3^	0.301 ± 0.084	15338 (491.539–5230)	12.72	0.0004	0.58	0.8468	1
Young—GHA ^4^	0.282 ± 0.101	19020 (430.750–8.599^10)	7.77	0.0053	1.618	0.0864	1.24
Old—NI8	0.311 ± 0.089	1617 (141.446–3692)	12.18	0.0005	0.629	0.0805	0.11
Old—GHA	0.124 ± 0.084	16780 (–)	2.18	0.1394	1.657	0.0764	1.09
Old—KUDSC-001 ^5^	0.249 ± 0.056	64.3885(7.599–720.2412)	19.66	<0.0001	1.423	0.1545	0.004

(–) Significant Regression was not obtained in Probit analysis. ^1^ Test for slopsignificance indicates dose affects mortality. ^2^ Test for Goodness of Fit (GoF) significance indicates error from Probit trend is greater than expected for simple binomial response. ^3^ NI8-Native Delta Nississippi Strain (ARSEF 8889). ^4^ GHA—Commercial strain. ^5^ KUDSC-001—South Carolina Kudzu Strain (ARSEF 13136).
